# Adjustment Disorder: Current Developments and Future Directions

**DOI:** 10.3390/ijerph16142537

**Published:** 2019-07-16

**Authors:** Meaghan L. O’Donnell, James A. Agathos, Olivia Metcalf, Kari Gibson, Winnie Lau

**Affiliations:** 1 Phoenix Australia Centre for Posttraumatic Mental Health, 161 Barry Street, Carlton VIC, Melbourne 3053, Australia; 2 Department of Psychiatry, University of Melbourne, Melbourne 3053, Australia

**Keywords:** adjustment disorder, review, diagnosis, symptoms, nosology, DSM-5, ICD-11, course, trajectory, treatment

## Abstract

Despite its high prevalence in clinical and consultant liaison psychiatry populations, adjustment disorder research has traditionally been hindered by its lack of clear diagnostic criteria. However, with the greater diagnostic clarity provided in the Diagnostic and Statistical Manual of Mental Disorders – fifth edition (DSM-5) and the International Statistical Classification of Diseases and Related Health Problems, 11th edition (ICD-11), adjustment disorder has been increasingly recognised as an area of research interest. This paper evaluates the commonalities and differences between the ICD-11 and DSM-5 concepts of adjustment disorder and reviews the current state of knowledge regarding its symptom profile, course, assessment, and treatment. In doing so, it identifies the gaps in our understanding of adjustment disorder and discusses future directions for research.

## 1. Introduction

Adjustment disorder describes a maladaptive emotional and/or behavioural response to an identifiable psychosocial stressor, capturing those who experience difficulties adjusting after a stressful event at a level disproportionate to the severity or intensity of the stressor [1]. The symptoms are characterised by stress responses that are out of step with socially or culturally expected reactions to the stressor and/or which cause marked distress and impairment in daily functioning. Unlike posttraumatic stress disorder (PTSD) or acute stress disorder (ASD), which have clear criteria for what constitutes a traumatic event, adjustment disorder criteria does not specify any requirements for what can be regarded as a stressor. Research has identified, however, that stressor events may include both traumatic events, such as exposure to actual or threatened death, as well as non-traumatic stressful events such as interpersonal conflict, death of a loved one, unemployment, financial difficulties, or illness of a loved one or oneself [2].

Prevalence estimates of adjustment disorder vary markedly due to various factors including sampling process, population, and the diversity of measures used for assessment and diagnosis. Population-based studies have found prevalence rates of less than 1%, which may be due to limitations of the diagnostic tools used [3]. Conversely, more recent studies using newer diagnostic tools have found prevalence rates of 2% in general population research [4]. Rates are much higher in specific high-risk samples such as recently unemployed (27%; [5]) and bereaved individuals (18%; [6]).

Adjustment disorder is particularly prevalent in consultation liaison settings [7]. A multisite study in consultation psychiatry services in the United States, Canada, and Australia found that adjustment disorder was diagnosed in 12% of psychiatric consultations, with a further 11% identified as possible cases [8]. In Irish general hospital patients, adjustment disorder represented 18.5% of consultation liaison referrals [7]. At least one psychosocial stressor was noted in 93% of all patients, which included medical illness in 59% of patients. In this setting, the diagnosis was used especially in patients with serious medical conditions, self-harm, injury and poisoning, and in cases presenting with a mixture of somatic and psychic symptoms. Other consultant liaison psychiatry samples have reported a prevalence rate as high as 30% [9]. In emergency department settings when routine psychiatric assessments have been conducted in individuals primarily presenting with self-harm, adjustment disorder was the most common diagnosis (32%; [10]). Among other medical populations, adjustment disorder is also extremely common. A 2011 meta-analysis of oncology-related palliative and non-palliative settings indicated a prevalence rate of 15–19%, comparable to major depressive disorder and higher than anxiety disorders [11]. Research from Japan shows the prevalence of adjustment disorder to be 35% among individuals with recurrent breast cancer [12]. In an acutely ill medical inpatient unit, adjustment disorder was found to be the most common diagnosis (14%), more than double the rates of depressive and anxious disorders [13].

Despite research indicating significant prevalence rates that are often greater than depressive and anxiety disorders in some populations, adjustment disorder has historically attracted little empirical research. Consequently, relatively little is known regarding the phenomenology of the disorder, its neural correlates, prevalence, risk factors, course, or treatment [14,15,16]. A key contributor to this lack of research has been the absence of clearly defined diagnostic criteria [15], which means operationalising the disorder in an empirical research context has proven difficult [17]. The adjustment disorder concept has attracted significant criticism due to issues related to its diagnostic vagueness. Research has struggled to neatly establish the extent to which adjustment disorder differs from other psychiatric disorders, or from normal adaptive stress responses [18].

Conceptualisation of adjustment disorder, however, is currently in a state of transition. With the most recent revisions of the two main diagnostic manuals used in clinical and research practice, the Diagnostic and Statistical Manual of Mental Disorders (DSM-5) [1] and International Statistical Classification of Diseases and Related Health Problems, 11th edition (ICD-11) [19], adjustment disorder has been increasingly recognised as an important target for research. The aim of this paper is to (i) compare and contrast the DSM-5 and ICD-11 diagnostic criteria for adjustment disorder; (ii) examine the course and trajectory of adjustment disorder; (iii) examine measurement of adjustment disorder; and (iv) discuss adjustment disorder treatment research. In doing so, this paper aims to identify gaps in our current knowledge of adjustment disorder and present directions for future research.

## 2. Diagnostic Criteria

The historical narrative for adjustment disorder in DSM and ICD has been described elsewhere [20,21] and provides a useful background to the current criteria. In DSM-5, adjustment disorder was reclassified to sit alongside PTSD and ASD in the *Trauma- and Stressor-Related Disorders* chapter [1]. Despite this, the diagnostic criteria remained effectively unchanged from the DSM-IV, as the committee decided that any proposed changes would be atheoretical given the lack of research that had been conducted into the disorder [14,17]. The focus of the DSM-5 approach to adjustment disorder has remained on distress or impairment associated with a stressor that is judged to be excessive (relative to cultural norms). On the other hand, the ICD-11 introduced changes that marked a significant paradigm shift. In line with DSM, ICD recognised adjustment disorder as a stressor related disorder by categorising it within the chapter *Disorders Specifically Associated with Stress*. It diverges from DSM by conceptualising adjustment disorder as a failure to adapt to a stressor as evidenced by preoccupation with the stressor and its consequences. Table 1 provides a summary of both DSM-5 and ICD-11′s diagnostic criteria for adjustment disorder.

### 2.1. Commonalities between DSM-5 and ICD-11

In their current iterations, the DSM-5 and ICD-11 diagnoses of adjustment disorder have many commonalities. Under both sets of criteria, a diagnosis of adjustment disorder must occur in the wake of an identifiable life stressor, and can only be diagnosed in the absence of another clinical diagnosis. Both systems recognise adjustment disorder as a transient condition, with DSM-5 stating that symptoms must not persist longer than six months after the stressor (and its consequences) are resolved, and ICD-11 recognising that symptoms tend to resolve within six months unless the stressor persists for a longer duration. Both additionally outline that emotional distress and functional impairments are key components of the disorder.

### 2.2. Differences between DSM-5 and ICD-11

The two sets of diagnostic criteria differ in key areas. The ICD-11 definition necessitates the identification of significant impairments in personal, occupational, and/or social functioning. Conversely, DSM-5 does not specifically require functional impairment—it is sufficient to have either impairments in functioning or distress that is disproportionate to the severity of the stressor. The ICD-11 also mandates that symptoms must emerge within one month of the stressor, while the DSM-5 allows a more liberal onset window of three months. Further, the DSM-5 specifies that symptoms cannot represent normal and culturally appropriate bereavement, whereas this is not mentioned by the ICD-11. However, the most significant difference between the diagnostic definitions is that ICD-11 requires symptoms of preoccupation with the stressor and its consequences in the form of rumination, excessive worry and/or recurrent distressing thoughts. DSM-5 gives no guidance as to what symptoms might constitute distress.

Overall, there is growing empirical support for the ICD-11 redefinition. Multiple studies investigating the diagnostic architecture of the disorder have identified items relating to stressor preoccupation and failure to adapt [4,22,23] which relate strongly to the core adjustment disorder concept. One longitudinal study over twelve months showed that intrusive memories was one of the symptoms that predicted adjustment disorder [17], supporting the ICD-11 idea that adjustment disorder is characterised by the mental intrusion of (and preoccupation with) the stressor.

‘Failure to adapt’ is thought to constitute a stress-response (e.g., sleep disturbances or concentration problems) that results in significant impairment in social, interpersonal, occupational, educational, or other areas of functioning [22]. Confirmatory factory analyses have shown that the two core symptoms of ICD-11 adjustment disorder (i.e., failure to adapt and preoccupations) comprise an accurate model of adjustment disorder symptom architecture, with high levels of model fit [23]. Four accessory symptoms (avoidance, depression, impulsivity, and anxiety) in addition to the core symptoms have also been found [4,23]. This suggests that in addition to the two core ICD-11 symptoms, there is evidence that additional symptoms may inform consideration of the diagnostic criteria.

#### 2.2.1. Subtypes

Another key point of difference between the two systems is that the ICD-11 has removed any reference to adjustment disorder subtypes, preferencing a unifaceted concept of adjustment disorder. Conversely, the DSM-5 delineates the disorder into a series of six subtypes, each signifying the presence of specific symptoms. DSM-5 differentiates between adjustment disorder with (1) depressed mood, (2) anxiety, (3) mixed anxiety and depressed mood, (4) disturbance of conduct, (5) mixed disturbance of emotions and conduct, and (6) unspecified [1]. Yet since the publication of DSM-5, there has been little evidence to support the idea of distinct subtypes of adjustment disorder [17]. In Glaesmer et al.’s [4] six-factor model of adjustment disorder—comprising factors related to preoccupations, failure to adapt, avoidance, depression, anxiety, and impulsivity—inter-correlations between each of the factors were extremely high (between 0.75 and 0.96), suggesting that these were not adequately distinguishable from each other. Given that many of these factors map directly onto the subtypes listed in the DSM-5 (where the ‘disturbance of conduct’ subtype is mirrored by the ‘avoidance’ and ‘impulsivity’ factors), the finding that these are so highly inter-correlated undermines the plausibility of distinct adjustment disorder subtypes. Indeed, this finding has been mirrored in more recent studies using both confirmatory factor analysis and bifactor modelling, which all found that group factors mapping onto DSM adjustment disorder subtypes were highly inter-correlated [23,24,25]. These findings collectively suggest that there is insufficient evidence at present to substantiate the existence of adjustment disorder subtypes, instead lending support to the unidimensional conception of adjustment disorder outlined in the ICD-11.

#### 2.2.2. Adjustment Disorder as a Subsyndromal Disorder

Both DSM-5 and ICD-11 adhere to the idea that adjustment disorder can only be diagnosed in the absence of another disorder. While most other disorders have the requirement that the symptoms cannot be better explained by another disorder, the adjustment disorder criteria are much more restrictive. As such, it is often conceived of as a subclinical or mild disorder. There is some evidence to suggest that this is indeed the case. In a longitudinal study of serious injury survivors, O’Donnell and colleagues found that across measures of disability, quality of life, anxiety and depression, those with adjustment disorder reported significantly worse outcomes than those with no disorder, but significantly better outcomes than those with another psychiatric diagnosis [17]. Consistent with this, DSM-5 explicitly instructs those presenting with subsyndromal PTSD to be diagnosed with adjustment disorder [1].

The fact that ICD-11 and DSM-5 have taken different approaches to a given diagnosis is not specific to adjustment disorder. Indeed, this issue has been raised in the PTSD literature, given the ICD and DSM nomenclature for PTSD are remarkably different [26]. The issue of whether treatments developed to treat the DSM-5 version of the disorder will be as effective in the treatment of its ICD-11 counterpart remains a challenge to optimising treatment for PTSD as it does for adjustment disorder [27]. Ultimately, while the differences between DSM-5 and ICD-11 adjustment disorder are significant, the divergence of ICD-11 in creating established clear, specific criteria for adjustment disorder has created a significant opportunity. The ICD-11 provides a description of the diagnosis that is much easier to operationalise than DSM-5, and consequently far more research has been conducted into ICD-11 adjustment disorder than DSM-5 adjustment disorder despite DSM-5 diagnosis being in situ since 2013. Since the introduction of the new ICD-11 diagnostic criteria in 2013, a scoping review conducted just three years later in 2016 found 10 new studies on international samples analysing the factor structure, measurement validity, risk factors, and outcomes from treatment intervention studies [28]. By establishing diagnostic criteria, the ICD-11 has given researchers the capacity to explore the research more clearly in a way that the vaguer structure in the DSM-5 does not permit. The ICD-11 proposal has allowed the adjustment disorder field to move ahead significantly.

## 3. Course and Trajectory

Research into the course of adjustment disorder is largely in its infancy. However, preliminary studies have identified that in some subpopulations, symptoms may increase over time, marking a trajectory toward a more severe disorder. In a study by O’Donnell et al. (2016), trauma survivors who had adjustment disorder 3 months after exposure were 2.67 times more likely to meet criteria for a more severe psychiatric disorder (including PTSD, major depressive disorder, and generalised anxiety disorder) at 12 months, relative to those who had no disorder at 3 months [17]. This finding runs counter to the proposal that adjustment disorder is a short-term diagnosis, with evidence suggesting that the disorder will progress to a more serious disorder in a subset of those diagnosed with adjustment disorder. Further, in this same study, 34.6% of those with adjustment disorder at three months still met the diagnostic criteria at twelve months suggesting a persistence of symptomatology.

**Figure 1:**
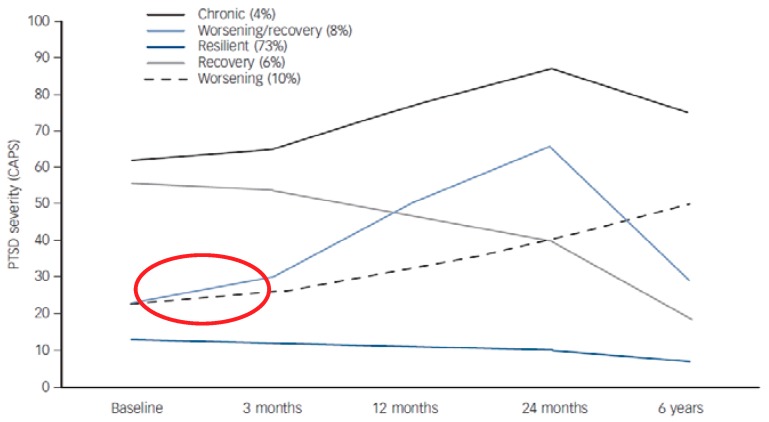
Posttraumatic stress disorder (PTSD) symptom trajectories over time. From Bryant et al. [37]. The red circle indicates the two trajectories of PTSD symptoms that may represent adjustment disorder trajectories.

Research into the course of PTSD may hold some answers to the trajectory of adjustment disorder over time. There have been a number of studies that have examined the trajectory of PTSD symptoms over time [29,30,31,32,33,34,35]. Generally, these studies show that the majority of those who are exposed to trauma typically fall into one of four to five prototypical trajectories (see Figure 1). It is reasonable to posit that those in the circled trajectories represent adjustment disorder given their initial response to the stressor is about 20 on the Clinician Administered PTSD Scale (CAPS; [36]) measure. A normal recovery is experienced by the majority of trauma survivors and is represented by the resilient group (whose initial CAPS score is approximately 10). The trajectories that start with a CAPS score of above 50 represent those with a probable PTSD diagnosis. It is interesting to note that both adjustment disorder trajectories accumulate symptoms over time, again suggesting that adjustment disorder is an early marker for a more severe disorder.

It is important to recognise that these trajectory analyses are within trauma samples (rather than a stressful events sample) so these adjustment disorder trajectories may represent the more severe end of the spectrum. It is also recognised that these trajectory analyses are more relevant to the DSM-5 construct of adjustment disorder rather than the ICD-11, because they do not include symptoms of rumination or worry. They do, however, provide a useful idea as to the course of adjustment disorder over time, suggesting that adjustment disorder in some populations may have an enduring course.

Although emerging evidence indicates that adjustment disorder is a gateway to more severe psychiatric disorders, it is important to highlight that adjustment disorder is associated with significant negative outcomes in and of itself. Consultant liaison psychiatry research indicates adjustment disorder is significantly associated with suicidality and self-harm, at similar proportions to depressive disorders [38]. Other studies in inpatient populations have likewise found rates of self-harm and suicidality are significantly higher in adjustment disorder compared to other diagnoses [39,40].

## 4. Assessment

As with most aspects of adjustment disorder, the development of adequate assessment tools historically has been hindered by the fact that diagnostic criteria for the disorder were not clearly specified. However, even now that the ICD-11 has somewhat remedied this shortcoming, there is a clear dearth of measures available for its assessment and diagnosis. Most general structured clinical interviews do not provide any level of assessment of adjustment disorder, with no diagnostic module in either of the Clinical Interview Schedule (CIS; [41]) or the Composite International Diagnostic Interview (CIDI; [42]). Those that do include one, such as the Scheduled Clinical Interview for DSM-5 (SCID; [43]) and the Mini International Neuropsychiatric Interview (MINI; [44]) administer only a few items relating to adjustment disorder, and only as an addendum if none of the diagnostic criteria for any other disorders are met. Naturally, this is in line with the ICD-11 and DSM-5 portrayals of adjustment disorder as a subthreshold disorder—however, these modules are typically too cursory to provide a methodologically adequate measure of adjustment disorder [16,45].

Recently, however, specific measures for adjustment disorder have begun to emerge. One such option is the Diagnostic Interview for Adjustment Disorder (DIAD; [46]), which is a structured clinical interview for adjustment disorder based on the DSM-5 criteria. The DIAD includes 29 items that aim to identify symptoms associated with a stressor, and evaluate the levels of distress and functional impairment associated with these symptoms. Preliminary attempts at validating the measure by the original authors suggested “moderate to good” concept and construct validity [46]. However, as yet there are no external attempts by other authors to validate the DIAD in any clinical trials or studies—it is therefore unclear to what extent the measure actually provides a valid index of adjustment disorder in a clinical context.

The Adjustment Disorder—New Module (ADNM) has been developed for the ICD-11 diagnosis of adjustment disorder, and is available as a structured clinical interview [47] or self-report questionnaire [2]. The first section asks participants to select from a list of stressors (acute and chronic life events) that have been present over the past year, and to identify which was the most prominent or distressing. The second section comprises 20 items, which form six subscales in accordance with ICD-11 criteria relating to pre-occupation, failure to adapt, avoidance, depressive mood, anxiety, and impulse disturbance. A longer-form version with 29 items also exists, but the ADNM-20 seems to be used more commonly [48]. Participants rate on a 4-point Likert scale how often they have experienced particular symptoms during the past two weeks, and overall symptom severity is calculated as a sum of all item scores. Attempts at validating the ADNM have yielded positive results, with studies suggesting good levels of diagnostic specificity and sensitivity [23,48,49]. Condensed forms of the ADNM, such as the ADNM-8 and ADNM-4, have also shown high levels of convergent and construct validity, suggesting these offer an alternative screening tool for assessing adjustment disorder symptoms which is equally valid, but briefer [50,51]. Ultimately, the ADNM and DIAD seem to provide the most comprehensive measures of the ICD-11 and DSM-5 concepts of adjustment disorder respectively, though further research is needed to validate the latter.

## 5. Treatment and Intervention

To date, there is only one published systematic review of treatments available for adjustment disorder. A 2018 review examined 29 treatment trials investigating current options for psychological and pharmacological intervention [52]. They found that the quality of evidence in these studies was “low” to “very low” according to Grading of Recommendations Assessment, Development and Evaluation (GRADE; [53]) guidelines. A key limitation to most of these studies was the lack of a measure of adjustment disorder, small sample sizes, and lack of follow-up assessments. The authors also raised the issue of the divergence of the ICD-11 and DSM-5 diagnostic classification. For example, the recent trial on self-help intervention was based on the beta version of ICD-11 and they found this intervention had its most useful impact on preoccupation about the event including rumination, worry and intrusive thoughts [54]. While this is very relevant for the ICD-11 diagnosis of adjustment disorder, as discussed earlier, the degree to which this would be useful for those meeting criteria for DSM-5 adjustment disorder is unknown.

Since the publication of the systematic review in 2018, two further randomised controlled trials (RCTs) have been published. One investigated an internet-based self-help intervention known as Brief Adjustment Disorder Intervention (BADI) for the treatment of ICD-11 adjustment disorder [55]. In the self-help trial, completer analysis revealed that BADI reduced ICD-11 adjustment disorder symptoms and increased psychological well-being for those participants who used the intervention at least once in 30 days. The high drop-out rates from this trial (86%) prevent firm conclusions from being drawn. A second study targeted ICD-10 and DSM-IV adjustment disorder, and compared a face-to-face and virtual reality delivered cognitive behavioural therapy (CBT) to the waitlist [56]. Both the face-to-face and virtual reality CBT resulted in significantly greater improvements to adjustment disorder relative to the wait-list controls at pre/post treatment. The virtual reality group had significantly greater longer-term improvements than the standard and wait-list groups. Despite very small sample sizes in this study, as well as the high drop-out rates from the Eimontas et al. study [55], there is early support that technology assisted interventions for adjustment disorder may be useful, though further methodologically rigorous studies are needed.

As adjustment disorder is characterised as a subclinical disorder, it is reasonable to consider that it may be responsive to lower intensity, brief intervention. This is consistent with intervention findings that show adjustment disorder to be responsive to self-help bibliotherapy [54], and other online self-directed interventions [55]. Adjustment disorder interventions might also be amenable to ‘task shifting’, that is, interventions designed to be delivered by non-specialists in order to increase their accessibility. Recent meta-analyses indicate that use of non-specialists can lead to significant improvements in mental health [57]. A recently developed program, Skills for Life Adjustment and Resilience (SOLAR), aims to address adjustment difficulties and sub-clinical presentations using a brief, non-specialist delivered format. The SOLAR program is currently being tested in Australia, Japan, and the South Pacific. So far, preliminary data drawn from these projects suggest that SOLAR is not only an acceptable and feasible intervention that can be implemented by trained lay workers, it is also effective in reducing adjustment difficulties [58,59].

In summary, the emergence of clear diagnostic criteria with ICD-11 has finally presented the opportunity for new treatment options to be developed and tested. Several emerging treatment options have utilised the internet to complement the therapeutic approach, which is likely to be appealing to individuals with sub-clinical problems such as adjustment disorder [56]. Additionally, treatments that are brief and scalable may be appropriate for the treatment of adjustment disorder. Despite this emerging evidence base, however, the lack of high quality trials that test interventions for adjustment disorder is still a serious concern, and there are no clear recommendations on how to best treat the disorder. As such, there is a clear need for higher quality, methodologically sound treatment trials to aid in both the development of new treatment options and in the validation of current ones.

## 6. Conclusions

After decades of uncertainty surrounding adjustment disorder, despite research indicating it is a prevalent problem in populations such as consultant liaison psychiatry, it is now a critical time for advancing our knowledge of the disorder. The establishment of clear diagnostic criteria in ICD-11 has produced a number of new studies, yet important questions remain about adjustment disorder—particularly around its phenomenology, course and treatment. Future endeavors might include a focus on emotional and behavioural correlates of adjustment disorder and mechanisms that underpin differences in symptom trajectory (e.g., how adjustment disorder may persist over time or develop into other psychiatric conditions), and how to build the evidence base for treatments designed or adapted for adjustment disorder. As adjustment disorder becomes increasingly legitimised and more clearly defined in the DSM and ICD, researchers in the psychiatric field have the ability to shed new light on a poorly understood disorder. In doing so, we can ensure that adjustment disorder patients have access to appropriate treatment and that clinical judgment is empirically informed.

## Figures and Tables

**Table 1:** Summary of corresponding DSM-5 [1] and ICD-11 [19] diagnostic criteria for adjustment disorder.

DSM-5	ICD-11
A. Onset of emotional or behavioural symptoms must occur in response to identifiable stressor, and within 3 months of the stressor.	1. Presence of an identifiable psychosocial stressor(s). Symptoms emerge within 1 month of the stressor.
B. These symptoms are clinically significant, marked by:	2. Preoccupation related to the stressor or its consequences in the form of at least one of the following:
- Distress that is disproportionate to the severity or intensity of the stressor, taking into account contextual and cultural factors.	(a) excessive worry about the stressor (b) recurrent and distressing thoughts about the stressor (c) constant rumination about the implications of the stressor.
or	
- Significant impairments in social, occupational or other domains of functioning.	3. Failure to adapt to the stressor that causes significant impairment in personal, family, social, educational, occupational or other important areas of functioning
C. The disturbance does not meet the diagnostic criteria for another mental disorder, and is not an exacerbation of a pre-existing disorder.	4. Symptoms are not of a sufficient specificity or severity to justify diagnosis of another mental or behavioural disorder.
D. The symptoms do not represent normal bereavement.	
E. Symptoms do not last for more than six additional months after the stressor or its consequences have been resolved.	5. Symptoms typically resolve within 6 months, unless the stressor persists for a longer duration
